# Supplementary material for: Acceptability of the FIGO Nutrition Checklist in Preconception and Early Pregnancy to Assess Nutritional Status and Prevent Excess Gestational Weight Gain: A Study of Women and Healthcare Practitioners in the UK
Source: Nutrients. 2022 Sep 1;14(17):3623. doi: 10.3390/nu14173623 (PMC9460608; doi:10.3390/nu14173623)
Supplement: Supplementary file 1 [file nutrients-14-03623-s001.zip › nutrients-1842710-supplementary.pdf]

**File S1:** FIGO nutrition checklist [15].

International Federation of Gynaecology and Obstetrics. The FIGO Nutrition Checklist; FIGO: London, UK, 2020. Available to download at: https://obgyn.onlinelibrary.wiley.com/action/downloadSupplement?doi=10.1002%2Fijgo.13321&file=ijgo13321-sup-0001-Supinfo.pdf (accessed on 14 July 2022).

**File S2.** Modified FIGO nutrition checklist.

# NUTRITION CHECKLIST FOR PRE-PREGNANT/ EARLY PREGNANT WOMEN

**Good nutrition in the mother, both before and during pregnancy, is important in ensuring healthy outcomes for her and her baby. In a clinical setting, the questions below would be completed by a woman in conjunction with her healthcare professional (e.g. midwife) to assess whether her nutritional intake is sufficient, and provide a basis for the health care professional to advise where changes need to be made (if applicable).**

## 1. DO YOU HAVE ANY SPECIAL DIETARY REQUIREMENTS (E.G. VEGETARIAN, VEGAN, ALLERGIES)? IF YES, PLEASE LIST BELOW:

..........................................................................................................................................................................................

## 2. WHAT IS YOUR:

a. Weight ........ kgs
b. Height ........... m
c. BMI ............ kg/m2 (Health care professional to complete): Divide weight in kg by height in metres then divide the answer by your height again to get your BMI.

## 3. QUALITY OF DIET

i) Do you eat meat or chicken 2-3 times per week? Yes / No

ii) Do you regularly eat more than 2 – 3 portions of fruit or vegetables per day? Yes / No

iii) Do you eat fish at least 1-2 times per week? Yes / No

iv) Do you consume dairy products (such as milk, cheese, yogurt) every day? Yes / No

v) Do you eat whole grain carbohydrate foods (brown bread, brown pasta, brown rice or other) at least once a day? Yes / No

vi) Do you consume packaged snacks, chocolates, biscuits, cakes, pastries or sugar-sweetened drinks less than 5 times a week? Yes / No

## 4. OTHER

i) If you are pregnant, did/do you take folate/folic acid supplements in pre-pregnancy and in early pregnancy (first 12 weeks)? Yes / No

ii) Do you get regular exposure to the sun (face, arms and hands for at least 10-15 mins per day)? Yes / No

iii) Has the doctor/nurse tested your haemoglobin (level of iron in the blood)? Yes / No
(Health care professional to complete) If yes, is it more than 110 g/l? Yes / No Enter the value: .................

## 5. EXERCISE

Do you exercise less than 3 times per week? Yes/ No
(this could be, for example, 30 minutes of brisk walking)

If you have answered No to any of the questions in section 3 or 4 your nutritional status may need to be assessed in more detail.

## ADDITIONAL DETAILS FOR THE HEALTHCARE PROVIDER

1. A healthy BMI is usually considered to be between 18.5–25 Kg/m2, although this depends on age and geographical region.

2. For women who are not pregnant, counsel on achieving a healthy weight before conceiving.

* For pregnant women provide indications for appropriate gestational weight gain according to pregravidic BMI (see right). This may vary according to local contexts.

| Pre-pregnancy BMI category | Total weight gain (kg) | Rate of weight gai 2nd and 3rd trimeste (kg/wk) |
|---|---|---|
| Underweight <18.5 kg/m² | 12.5 - 18 | 0.51 (0.44 – 0.58 |
| Normal weight 18.5-24.9 kg/m² weight | 11.5 - 16 | 0.42 (0.35 – 0.50 |
| Overweight 25.0 – 29.9kg/m² weight | 7-11.5 | 0.28 (0.23 – 0.33 |
| Obese >30kg/m² | 5-9 | 0.22 (0.17 – 0.27 |

*From 2009 Institute of Medicine guidelines on gestational weight gain: https://www.nationalacaden org/hmd/~/media/Files/Report%20Files/2009/Weight-Gain-During-Pregnancy-Reexamining-the-Guidelines/Report%20Brief%20-%20Weight%20Gain%20During%20Pregnancy.pdf*

3. Q 3. i. is to assess whether vitamin B12, iron and protein intake is sufficient.
4. Q 3. ii. is to assess whether intake of antioxidants, micronutrients and fibre is sufficient.
5. Q 3. iii. is to assess whether intake of omega 3 / omega 6 polyunsaturated fatty acids, vitamin D and iodine is sufficient.
6. Q 3. iv. if the patient answers No to this question, calcium supplementation should be considered.
7. Q 3. v. and vi. – if No, discuss how the woman could increase intake wholegrains and reduce processed sugar in their diet.
8. Q 4. i. if not taking a folate supplement suggest a folate supplement (dosage below)
9. Q 4. ii. if the patient has little sun exposure or has dark skin, remind them about the recommended Vitamin D supplementation in pregnancy.
10. Q 4. Iii. if Hb < 110 g/l suggest an iron supplement. This cut off may vary according to local contexts.
11. Health care professionals should consider any foods available in their country which are considered unsafe for pregnancy.
12. As well as the questions in the questionnaire, health care professionals should assess whether any other potential unsafe aspects of the woman's lifestyle should be counselled on, such as smoking, alcohol, recreational drug use, or lack of physical exercise

## PROFESSIONALS INVOLVED: MIDWIVES, OBSTETRICIANS AND GYNAECOLOGISTS, GENERAL PRACTITIONERS, NUTRITIONISTS/ DIETICIANS, COMMUNITY HEALTH WORKERS, NURSES.

### PRE-PREGNANCY – WHEN PLANNING A PREGNANCY

**Assessment considerations**

-Diet Composition
-Physical activity
-Height, weight, BMI
-Obesity risk (waist circumference and other anthropometric measures)
-Anemia
-Risk of specific nutritional problems:
-Folate, Iron, Calcium, Vitamin B12, Vitamin D

**Discussion points**

-Importance of a healthy diet and exercise
-Problems of sedentary behaviour
-Risky behaviours and exposures – Tobacco, alcohol, recreational drugs, environmental toxins
-Chronic disease screening and management
-Supplementation folic acid (400mcg for all women preparing for pregnancy, and 5mg daily for omen at higher risk of NTDs (e.g. BMI>30, diabetes and epilepsy)
-Other nutrients (Iron, Iodine, Vitamin B12)

### DURING PREGNANCY

**Assessment considerations**

- Diet Composition
- Physical activity
- Height, weight, BMI
- Obesity risk (waist circumference and other anthropometric measures)
- Gestational Weight Gain
- Blood Pressure
- Risk of specific nutritional problems (low nutrient density or high calorie intake)
- Deficiencies from specific diets or under nutrition
- First Trimester: Folate, Vitamin B12, Iodine, PUFAs
- Second and Third Trimesters: Iron, Iodine, Calcium, folate, B vitamins, Vitamin D
- Energy intake (+200 Kcal/day), PUFAs

**Discussion points**

Dietary counselling
Safe levels of exercise
Sedentary time
Weight management and gestational weight gain
Risky behaviours and exposures – Tobacco, alcohol, recreational drugs, environmental toxins, sources of food borne infections
Pregnancy complications screening and management (GDM, Blood pressure)
Supplementation:
- Folic acid (400mcg through first 12 weeks)
- vitamin D supplement (10 micrograms per day)
- Other nutrients as required

**THIS DOCUMENT HAS BEEN ADAPTED FROM THE FIGO NUTRITION CHECKLIST BASED ON UK NUTRITIONAL GUIDELINES FOR PREGNANCY AND PRECONCEPTION**

**File S3.** Questionnaire for women.

Full list of questions listed here, however, on the online platform the survey appeared different as a logic was introduced so women only needed to answer questions relevant to their reproductive group.

**FIGO Healthy mother, healthy baby—Nutrition checklist for pre-pregnant / early pregnant/ post-partum women**

**Consent statement: Thank you for reading this information sheet and considering taking part in this research.**

☐ Please tick (check) this box to indicate that you have read and understood the information on this form,

are aged 18 or over and consent to take part in this survey.

**Section A: Personal details**

**None of the questions are compulsory/ mandatory and you can choose to omit answering questions that you may feel are uncomfortable.**

**i. Age:** ________________________

**ii. Post code:** ________________________

(Any personal identifiable information such as postcode you provide will be analysed anonymously in conjunction with your survey responses. We will not pass on these details directly to anyone or use it for identifying a person. Your postcode is needed to classify which region you live in. The survey results will be processed in adherence to GDPR)

**iii. In what region do you live?** *[choose one only]*: 1. East of England 2. East Midlands 3. London 4. Northeast 5. Northwest 6. Northern Ireland 7. Scotland 8. Southeast 9. Southwest 10. Wales 11. West Midlands 12. Yorkshire / Humberside

**iv. What is the highest level of education you have completed?** *[choose one only]* 1. Primary school 2. Secondary school up to 16 years 3. Higher or secondary or further education (A-levels, BTEC, etc.) 4. College or university 5. Post-graduate degree 6. Prefer not to say 7. Other

**v. Which of the following best describes your race or ethnicity?** *[choose one only]* 1. White 2. Black/Black British 3. Asian/Asian British 4. Mixed 5. Other 6. Prefer not to say

**vi. Are you pregnant now?** Yes No

If yes,

**Weeks of pregnancy:** ___________________________

**vii. Are you planning to get pregnant in the coming year and have visited a healthcare professional for advice related to pregnancy planning?** Yes No

**viii. Did you deliver a baby in the last 2 years?** Yes No

Have you visited a healthcare professional for advice related to pregnancy planning?

If you answered "yes" to the question above, whom did you consult or visit to discuss preparing for pregnancy? (e.g., GP, midwife, pharmacist) (open text response)

**Please complete Section B AFTER reading the FIGO Healthy mother, healthy baby – Nutritional questionnaire for pre-pregnant / early pregnant women (answer the questions as appropriate for your own diet and lifestyle)**

*(note – "We would like you to only read the full checklist. You do not need to complete the answers for the checklist. The aim of the checklist is to help women discuss nutritional issues with their healthcare provider. If you were in a clinic, this would be provided to you in the waiting room before your consultation. If you would like to keep the information on the checklist for your records, a link will be provided at the end of the survey to download it. All information on the checklist is in accordance with UK health guidelines")*

**Section B: Views on the FIGO Nutritional Checklist**

*Please tick the response that best characterises how you feel about each statement*

| | **Strongly Agree** | **Agree** | **Neutral** | **Disagree** | **Strongly Disagree** |
|---|---|---|---|---|---|
| The checklist will be easy to complete in a waiting room of a GP clinic/ Midwife's clinic | 5 | 4 | 3 | 2 | 1 |
| The checklist did not take too much time to read and understand | 5 | 4 | 3 | 2 | 1 |
| The checklist is easy to read | 5 | 4 | 3 | 2 | 1 |
| I have already thought about my diet for pregnancy before reading the checklist | 5 | 4 | 3 | 2 | 1 |
| I completed a similar checklist before and/or during pregnancy<br><br>If yes – where? (e.g., GP clinic, Midwife consultation) open ended answer | Yes | No | Cannot remember | | |
| The checklist contains useful information | 5 | 4 | 3 | 2 | 1 |
| I would recommend the use of the checklist for other women during pregnancy | 5 | 4 | 3 | 2 | 1 |
| **If you are planning another pregnancy, how much do you agree with the following statements: (for women who answered Yes to viii OR ix):** | | | | | |
| Changes to my diet before I become pregnant can help make sure my child is born healthy | 5 | 4 | 3 | 2 | 1 |
| If you had a baby in the last 2 years<br>How much do you agree/ disagree with the following statement | | | | | |
| I have tried to make changes after my last pregnancy (e.g., lose weight, eat more healthily | 5 | 4 | 3 | 2 | 1 |
| After my previous pregnancy, I received support with nutrition and weight related issues from healthcare workers | 5 | 4 | 3 | 2 | 1 |

**Section C:**

**During a consultation, you would be able to discuss the information on page 2 of the checklist and your answers with the healthcare professional (HCP)**

**When you were in hospital/ clinic did any healthcare professional (Doctor or midwife) discuss diet and nutrition with you?**

☐ Yes ☐ No Don't remember

**1.** With whom did you discuss this? GP ☐ Doctor ☐ Midwife Other ……….

For each of the statements below, please circle the response that best characterises how you feel about the statements that apply to your conversation(s) with the healthcare practitioner:

| | **Strongly Agree** | **Agree** | **Neutral** | **Disagree** | **Strongly Disagree** |
|---|---|---|---|---|---|
| The HCP had enough time to talk about my diet with me | 5 | 4 | 3 | 2 | 1 |
| Discussing my food habits and nutrition with the HCP was easy | 5 | 4 | 3 | 2 | 1 |
| I felt that I had the necessary information to improve my diet/ lifestyle | 5 | 4 | 3 | 2 | 1 |
| I feel better prepared to adopt a healthier lifestyle in pregnancy (after reading this checklist) | 5 | 4 | 3 | 2 | 1 |
| Nutrition has been a regular topic of discussion in my pregnancy | 5 | 4 | 3 | 2 | 1 |
| The FIGO checklist will improve discussions of nutrition with an HCP | 5 | 4 | 3 | 2 | 1 |

**2.** How would you feel if nutrition diet and weight gain during pregnancy were discussed routinely in all antenatal appointments (even if it was not the main reason for your visit)?

______________________________________________________________________

______________________________________________________________________

**3.** Do you suffer from any conditions requiring a special diet which was not addressed in the checklist? (e.g., coeliac disease)

Yes No

If yes, please add details here - ______________________________________________

We welcome any further comments and suggestions to improve the checklist

______________________________________________________________________

______________________________________________________________________

______________________________________________________________________

If you would like to save a copy of the checklist, complete it for your records or discuss with your practitioner you can download it here (link to checklist)

If you would like to be included in the prize draw for a £20 Amazon voucher, please add your email address here:
You will only be contacted if you have won the prize draw. Your contacts will be deleted after the study is completed and you will not be contacted in the future.

**File S4.** Questionnaire for HCPS.

**Consent statement: Thank you for reading this information sheet and considering taking part in this research.**

☐ Please tick (check) this box to indicate that you have read and understood the information on this form, are aged 18 or over and consent to take part in this survey.

**FIGO Healthy mother, healthy baby—Nutrition checklist for pre-pregnant / early pregnant/ post-partum women: acceptability questionnaire for UK Healthcare Practitioners**

**Section A: Demographic**

**1.** What is your staff category?

☐ Consultant OBGYN ☐ General Practitioner

☐ Community Midwife ☐ Staff Midwife ☐ Dietitian

☐ Other ….

**2.** How many years of clinical experience do you have?

☐ Less than 2 years ☐ 2-5 years ☐ 6-10 years

☐ More than 10 years ☐ Currently training

**3.** **Region of practice in the UK**

*[choose one only]*: 1. East of England 2. East Midlands 3. London 4. Northeast 5. Northwest 6. Northern Ireland 7. Scotland 8. Southeast 9. Southwest 10. Wales 11. West Midlands 12. Yorkshire / Humberside

**Section B: FIGO Nutrition Checklist**

**Please read both pages of the Nutrition checklist before answering the following questions. You do not need to complete the questions on the checklist. You will only need to read the checklist and complete the questions below). In a clinical setting, women will be expected to complete the checklist in the waiting room (page 1) and then discuss the findings with the healthcare practitioner.**

**4.** For each of the statements below, please circle the response that best characterises how you feel about the statement:

| | **Strongly Agree** | **Agree** | **Neutral** | **Disagree** | **Strongly Disagree** |
|---|---|---|---|---|---|
| The checklist will be easy to use | 5 | 4 | 3 | 2 | 1 |
| The checklist is easy to read (style and format) | 5 | 4 | 3 | 2 | 1 |
| The checklist is useful (content) | 5 | 4 | 3 | 2 | 1 |
| I will have enough time to go through the checklist in the clinic | 5 | 4 | 3 | 2 | 1 |

| | | | | | |
|---|---|---|---|---|---|
| The checklist can help initiate a discussion about nutrition in pregnancy | 5 | 4 | 3 | 2 | 1 |
| I will be able to discuss nutrition with women who have come for another issue/ routine check-up if they complete the questionnaire | 5 | 4 | 3 | 2 | 1 |
| I would use the checklist as a resource for nutrition health promotion in routine care | 5 | 4 | 3 | 2 | 1 |
| I would encourage women to use the checklist as a tool to assess their nutritional intake in pregnancy | 5 | 4 | 3 | 2 | 1 |
| The checklist covers important and common nutritional issues during pregnancy | 5 | 4 | 3 | 2 | 1 |

**5.** Are there other tools that you are currently using in **routine** practice to discuss nutrition during pregnancy? ☐ Yes ☐ No
If yes, please provide details: ______________________________
______________________________
______________________________

**6.** Do you think the checklist needs to be improved or changed to make it more effective in your practice?
☐ Yes ☐ No
If yes, please provide details on what can be modified: ______________________________
______________________________
______________________________

**Nutrition Discussion**

**7.** For each of the statements below, please select the response that best characterises how you feel about the statement:

| | **Strongly Agree** | **Agree** | **Neutral** | **Disagree** | **Strongly Disagree** |
|---|---|---|---|---|---|
| Discussions about nutritional intake are important for women **before** pregnancy | 5 | 4 | 3 | 2 | 1 |
| Discussions about nutrition are difficult to initiate in clinical practice | 5 | 4 | 3 | 2 | 1 |
| Discussions about gestational weight gain are difficult to initiate in clinical practice | 5 | 4 | 3 | 2 | 1 |
| I am confident in discussing nutrition with women | 5 | 4 | 3 | 2 | 1 |

| | | | | | |
|---|---|---|---|---|---|
| I am confident in discussing weight management with women | | | | | |
| I feel I have the necessary tools/ training needed for discussing nutrition and weight management with women | 5 | 4 | 3 | 2 | 1 |

**8.** For each of the statements below, please circle the response that best characterises how you feel about the statement:

| | **All the time** | **Most of the time** | **Sometimes** | **Occasionally** | **Never** |
|---|---|---|---|---|---|
| I currently discuss nutrition with women during routine antenatal/postnatal care | 5 | 4 | 3 | 2 | 1 |
| I initiate nutrition conversations during routine antenatal care | 5 | 4 | 3 | 2 | 1 |
| Women initiate conversations about nutrition during routine perinatal care | 5 | 4 | 3 | 2 | 1 |

**9.** What are the issues (if any) that you face while supporting women with dietary behaviour change or prevention of obesity? .

***10.*** *We welcome any further comments or suggestions for the checklist:*

---

**If you would like to save a copy of the checklist you can download it here**

If you would like to be included in the Prize draw for a £20 Amazon voucher, please add your email address here: ….

You will only be contacted if you have won the prize draw. Your contacts will be deleted after the study is completed and you will not be contacted in the future

*Thank you very much for completing this questionnaire. We appreciate your time.*

**File S5.** Categories from analysis for improving the content and use of the FIGO nutrition checklist in clinical practice.

| Category | Sub-categories (n) Women's survey | Sample quotes |
|---|---|---|
| **A. Gaps Identified** | • Eating Disorders (2)<br>• Inadequate consideration of Vegan/Vegetarian diets (9)<br>• Consideration of different trimesters and pre-pregnancy (2)<br>• Consideration of underweight (2) | "Disordered eating plays a significant role in nutritional choices, which is often in conflict to that of nutritional guidance for pregnant woman." (ID 1006) |
| **B. Recommendations for additional topics and revisions** | • Including information on High-risk pregnancies and other conditions (12)<br>• Portion size (2)<br>• Include Alcohol, smoking, caffeine (2)<br>• BMI measurement difficult (4)<br>• Exercise (3) | "During my first pregnancy, the midwife couldn't advise on whether I could continue the exercise regime I was doing because she didn't know… you are advised of everything you can't do … but not what you can actually do" (ID 1050) |
| **C. Additional support after completion of checklist** | • Empower women to make healthy dietary choices over routine screening (3)<br>• Provide evidence-based resources (11)<br>• Holistic care for women (1) | "I believe more supportive tools to take away from the meeting along with the communications provided at the meetings." (ID 1044) |
| **D. Effective and sensitive Communication** | • Checklist can support conversations during consultation (6)<br>• Information needs modification/ simplification (10)<br>• Sensitive and non-judgmental conversations needed (weight, BMI, nutrition and exercise) (12) | "It is just as important as the 'do not smoke or drink' message that women are told about throughout pregnancy, however, is not spoken about as much. It is definitely something that is often overlooked." (ID 1040)<br>"Any advice regarding nutrition and exercise should be offered in a non-pushy way. I feel the majority of women are well aware of the benefits of a healthy diet and exercise for a healthy pregnancy and should not be made to feel guilty if they do not happen to meet the perfect criteria at the time of pregnancy." (ID 1070) |
| **E. Additional points raised** | • BMI unreliable marker (5)<br>• HCP's level of knowledge (4) | "I do not believe that most healthcare professionals are well versed enough in plant-based diets to support the follow-up conversations". (ID 1117) |

*n* = number of coded comments; HCP, health care professional.
